# Effect of Commercially Available Sugar-Sweetened Beverages on Subjective Appetite and Short-Term Food Intake in Girls

**DOI:** 10.3390/nu10040394

**Published:** 2018-03-23

**Authors:** Lorianne J. Bennett, Julia O. Totosy de Zepetnek, Neil R. Brett, Kelly Poirier, Qing Guo, Dérick Rousseau, Nick Bellissimo

**Affiliations:** 1 Department of Applied Human Nutrition, Mount Saint Vincent University, 166 Bedford Highway, Halifax, NS B3M 2J6, Canada; loriannebennett@gmail.com (L.J.B.); poirierkelly@hotmail.com (K.P.); 2 Faculty of Kinesiology & Health Studies, University of Regina, 3737 Wascana Parkway, Regina, SK S4S 0A2, Canada; julia.totosy@uregina.ca; 3 School of Nutrition, Ryerson University, 350 Victoria Street, Toronto, ON M5B 2K3, Canada; neil.brett@ryerson.ca; 4 Department of Chemistry & Biology, 350 Victoria Street, Toronto, ON M5B 2K3, Canada; qing.guo@ryerson.ca (Q.G.); rousseau@ryerson.ca (D.R.)

**Keywords:** girls, food intake, appetite, sugars, cola, chocolate milk, fruit drink

## Abstract

Background: The effect of sugar-sweetened beverages (SSBs) on satiety and short-term food intake (FI) regulation in girls has received little attention. The objective of the present study was to compare the effect of pre-meal consumption of commercially available SSBs on subjective appetite and short-term FI in 9–14-year-old girls. The methods we used include using a randomized crossover design in which 28 girls consumed isovolumetric amounts (350 mL) of a fruit drink (154 kcal), cola (158 kcal), 1% chocolate milk (224 kcal), or water (control; 0 kcal) on four separate mornings. Subjective appetite and thirst were measured at regular intervals via visual analogue scales (VAS) and FI was assessed at 60 min post-beverage consumption. The results show that subjective appetite and thirst decreased after all beverages, but did not differ among beverages. Short-term FI was suppressed following consumption of chocolate milk (15%; *p* < 0.001) and cola (11%; *p* = 0.02) compared to the water control. However, cumulative energy intake (beverage (kcal) + test meal (kcal)) was not affected by beverage type. In conclusion, chocolate milk and cola, but not fruit drink, suppressed FI in girls while cumulative FI did not differ among treatments.

## 1. Introduction

Sugar-sweetened beverages (SSBs) have become readily available sources of energy [1,2], which has led to their increased contribution to daily energy intake among children [3]. SSBs account for up to 18% of daily energy intake and are consumed twice as much by children ≤10 years old compared with children >10 years old in Canada and the US [3,4]. Observational studies evaluating the association between consumption of SSBs and childhood obesity [5,6] suggest SSBs bypass food intake (FI) regulatory mechanisms leading to overconsumption [7]. Beverage guidance in North America suggests consuming primarily water followed by low-calorie nutrient-dense beverages such as milk and limiting SSBs [7,8,9]. However, a review of current recommendations for sugars intake found recommendations are based on low quality evidence [10]. Therefore, the effect of sugars on short-term FI regulation, especially among children, has been deemed one of the high-priority research gaps that should be addressed to inform dietary guidelines and policies [11].

It is unclear how sugars composition of beverages affects satiety and FI especially in children. Girls may experience greater satiety after sugar-sweetened beverage (SSB) intake compared to boys [12]. Fructose is thought to favor lipogenesis and bypass FI regulatory mechanisms. Additionally, observational studies in adults have associated high fructose corn syrup (HFCS) intake with increasing rates of obesity [13]. In contrast, glucose may suppress FI compared to other sugars. In healthy boys aged 9–14 years, FI was suppressed 60 min post-consumption of a glucose solution but not after sucrose or HFCS [14]. Conversely, short-term experimental studies in young adults reported similar effects of ratios of glucose to fructose in solution on subsequent FI 80 min post-ingestion [15] and found no difference in FI at 50 min [16] or in 24 h [17] glucose, insulin, leptin, and ghrelin levels when comparing consumption of fructose and sucrose.

It is unclear how energy content, macronutrient composition, and meal timing affect FI regulation in girls. There have been reports in healthy young adults showing no difference in test meal FI after pre-meal consumption of isocaloric beverages of orange juice, cola, or 1% milk [18]. Another study reported that consuming pre-meal isovolumetric beverages of 1% chocolate milk and infant formula resulted in decreased subsequent FI, compared to water, at 30 min but not at 2 h in healthy young adults [19]. Conversely, in the only previous report in children on the role of commercially available SSBs on FI regulation, chocolate milk increased satiety compared to a sugar-sweetened fruit drink, which resulted in about a 12% reduction in FI at a test meal 60 min later when compared to water in boys and girls 9–14 years of age [12]. Furthermore, there may be sex differences in FI regulation with girls having more restrained eating behaviors and restrictive FI [20]. Therefore, the objective of the present study was to compare the effects of consuming pre-meal isovolumetric SSBs varying in energy content and macronutrient composition (fruit drink, cola, 1% chocolate milk, water) on short-term FI and subjective appetite in 9-year-old to 14-year-old girls.

## 2. Materials and Methods

### 2.1. Participants

Twenty-eight girls aged 9–14 year were recruited by word of mouth and through newspaper advertisements. This study was approved by the Research Ethics Board at Mount Saint Vincent University. Body mass index percentile for age was assessed according to CDC growth charts [21]. Inclusion criteria were girls born at full-term, normal birth weight, and lactose tolerant. Exclusion criteria included girls who were dieting, taking medications that would affect study outcomes, and any significant learning, behavioral, or emotional difficulties. A telephone screening was used to assess eligibility while a screening session at the Department of Applied Human Nutrition at Mount Saint Vincent University was used to acquire informed written consent from parents and assent from children.

### 2.2. Experimental Design

This was a randomized, repeated-measures study where participants came for five laboratory sessions. The first session was a screening visit where consent, anthropometrics, and body composition were obtained. Participants were familiarized with study protocols and rated their pizza preferences. The four test sessions were scheduled at least one week apart. After a 10–12 h overnight fast, a standardized breakfast (290 kcal) of skim milk (250 mL, Baxter, Saint John, NB, Canada), breakfast cereal (26 g, Honey Nut Cheerios General Mills, Mississauga, ON, Canada), and orange juice (236 mL, Tropicana Products Inc., Bradenton, FL, USA) was consumed at home. Participants were instructed to not consume any other food between breakfast and the treatment beverage 2 h later. The start time of sessions was always 10:00 am or 11:00 am and was consistent within participants across the four test sessions.

Upon arrival to the lab, participants answered questions about whether they consumed the entire breakfast and if other foods were consumed prior to arrival. Participants were only allowed to continue with the study sessions if they consumed the entire breakfast. Afterward, participants consumed one of four test beverages within five minutes, which includes isovolumetric (350 mL) beverages of a fruit drink (154 kcal, Fruite^®^, A. Lassonde Inc., Montreal, QC, Canada), cola (158 kcal, Coca Cola^®^, Toronto, ON, Canada), chocolate milk (224 kcal, Baxter’s^®^ 1% M.F. Chocolate Milk, Saint John, NB, Canada), or a water control (0 kcal; Nestle Pure Life^®^, Toronto, ON, Canada). Table 1 details the nutritional composition of each beverage. Beverages were served in opaque cups with lids and straws and served chilled.

Sixty minutes following beverage consumption, girls were given an *ad libitum* pizza lunch and were instructed to eat until comfortably full. The pizza consisted of two varieties of Deep ‘N Delicious 5” pizza (pepperoni and three-cheese; donated by McCain Canada Ltd., Florenceville, NB, Canada). The girls participated in age-appropriate sedentary activities such as reading, doing homework, or completing crossword puzzles during the 60 min between beverage consumption and the *ad libitum* pizza lunch. The activities as well as the pizza preferences were standardized within each participant across all sessions. Questionnaires were administered at baseline, throughout the test session, and immediately following meal consumption.

### 2.3. Experimental Procedures

#### 2.3.1. Anthropometrics and Body Composition

Height (m) and mass (kg) were measured from which body mass index (BMI, kg/m^2^, and percentile) was calculated using the CDC growth charts [21]. Using a Harpenden skinfold caliper, skinfold measurements (mm) were taken by a trained technician to the nearest 0.1 mm at the triceps, biceps, suprailiac, and subscapular (Cambridge Scientific Industries, Cambridge, MD, USA) [23]. Age and sex-specific body fat percentage was estimated using the mean of three consecutive skinfold measurements and specific regression equations [24] from which fat mass (FM, kg) and fat free mass (FFM, kg) were estimated.

#### 2.3.2. Food Intake

Nutrient content of the pizza, cooking methods, and advantages of using the pizzas have been previously reported [14,25]. Each participant received three pizzas in 10 min intervals, sliced in quarters, and were instructed to eat until ‘comfortably full.’ Pizza consumption and nutrient intake was calculated using nutritional composition information and the weight of the pizza. The bottled water (Nestle Pure Life^®^, Toronto, ON, Canada) was weighed before and after the test meal to calculate water intake.

To calculate cumulative energy intake (kcal), the energy consumed from the pre-meal beverage and pizza were added. Using the following formula, ((FI after water control (kcal) − FI after beverage (kcal))/(kcal in beverage) × 100) [26,27], caloric compensation (CC) was calculated. CC scores <100% demonstrated participants did not fully compensate at the test meal for preload calories while CC scores >100% demonstrated an overcompensation for preload calories by eating less energy during the meal [26].

#### 2.3.3. Subjective Appetite

Visual analogue scales (VAS) assessed the effects of the beverage treatments on motivation to eat as well as measured physical comfort, sweetness, and pleasantness of the beverages along with pizza acceptability [26,28,29]. On each 100 mm VAS, participants marked an “X” on the line to indicate feelings between opposing statements anchored on either end [26]. Scores were determined by measuring the distance (mm) from the beginning of the line to the “X”.

To assess subjective appetite, a motivation to eat VAS included the following four questions: (1) desire to eat (“very weak” to “very strong”), (2) hunger (“not hungry at all” to “as hungry as I’ve ever felt”); (3) fullness (“not full at all” to “very full”), and (4) prospective consumption (“nothing at all” to “a large amount”). These questionnaires have previously been validated in children [28]. The following formula was used to calculate subjective average appetite ((desire to eat) + (hunger) + (100 − fullness) + (prospective food consumption)/4). VAS questionnaires were given at baseline (0 min), and at 15 min, 30 min, 45 min, 60 min, and after meal consumption (90 min).

### 2.4. Statistical Analyses

The effect of beverage treatment (fruit drink, cola, 1% chocolate milk, water) on FI, cumulative energy intake, water intake, sweetness, and pleasantness were tested via one-factor ANOVA. Change from baseline subjective average appetite scores were analyzed using a two-factor repeated measures ANOVA to assess the effects of beverage treatment and time. The Tukey-Kramer post hoc test was used to adjust for multiple comparisons and statistical significance was defined as *p* < 0.05. Data are reported as mean ± standard error of the mean (SEM). Statistical Analysis Software version 9.2 (SAS Institute Inc., Carey, NC, USA) was used to perform all statistical analyses.

## 3. Results

All twenty-eight girls completed the study. Girls ranged from being at a healthy weight to being obese, according to BMI percentiles. However, all data was pooled because there was no body mass status by beverage treatment interaction. Baseline characteristics are summarized in Table 2.

### 3.1. Food Intake, Water Intake, and Caloric Compensation

There was a main effect of beverage treatment (*p* < 0.001) on FI (see Table 3). FI was lower after chocolate milk (*p* < 0.001) and cola (*p* = 0.021) consumption when compared to water consumption. Fruit drink had no effect on FI when compared to the other beverages (*p* = 0.12–0.78). Cumulative FI was not affected by beverage treatment. Neither caloric compensation nor water intake were affected by beverage treatment (see Table 3).

### 3.2. Subjective Ratings from Visual Analogue Scales

**Figure 1:**
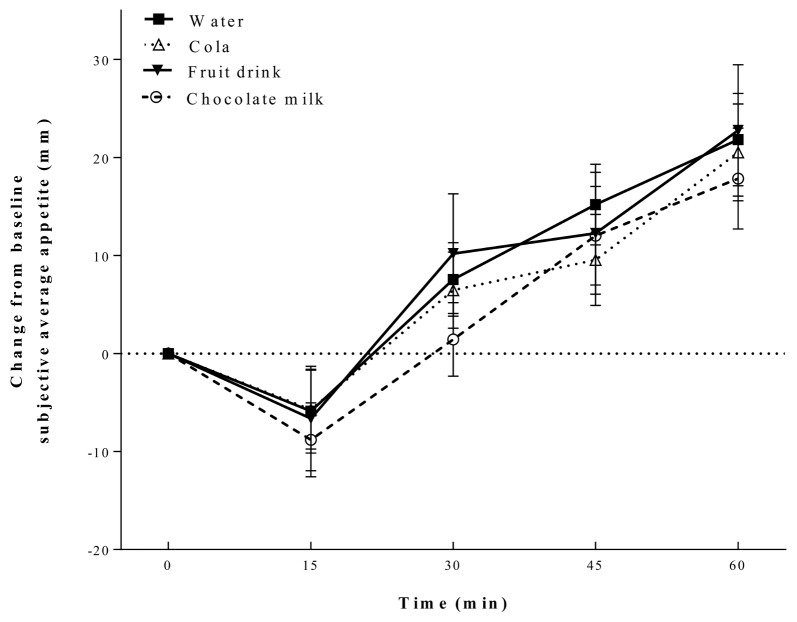
Change from baseline subjective average appetite. Appetite was affected by time (*p* < 0.001), but not by beverage or time by beverage interaction. All values are mean ± SEM (*n* = 28).

Change from baseline average appetite was affected by time (*p* < 0.001), but not beverage treatment (*p* = 0.65) and there was no beverage by time interaction (*p* = 0.99) (see Figure 1). Average appetite decreased after all beverages consumed at 15 min and then increased to 60 min. Change from baseline average subjective thirst was not affected by time or beverage. Additionally, there was no treatment by time interaction.

There was a main effect of beverage sweetness (*p* < 0.001) and pleasantness (*p* < 0.001). All beverages were rated sweeter than water (*p* < 0.001) and the fruit drink was rated sweeter than cola (*p* = 0.013). Both the fruit drink and chocolate milk were rated more pleasant than water (*p* < 0.001) and the chocolate milk was rated more pleasant than cola (*p* < 0.001). Pleasantness of the test meal was not affected by beverage treatment.

## 4. Discussion

The present study aimed to determine the effect of pre-meal SSB consumption on short-term FI regulation in young girls. The major findings suggest that both energy content and macronutrient composition of pre-meal SSBs influences short-term FI, but it may not influence cumulative energy intake. Both 1% chocolate milk (224 kcal) and cola (158 kcal) but not the fruit drink (154 kcal) reduced test meal FI compared with the water control. However, none of the pre-meal SSBs increased cumulative energy intake (SSB + test meal kcal) compared with water. Our findings indicate that consuming SSBs may not lead to increased FI in healthy young girls, which is contrary to previous reports suggesting SSBs lead to caloric overconsumption. In addition, while subjective average appetite decreased after all test beverages, it did not predict FI at the test meal.

Failure of the fruit drink to significantly suppress test meal FI despite its similar energy and sugars content with cola stands in contrast with several previous reports. It has been suggested that greater ratios of glucose to fructose found in the fruit drink may suppress FI more than higher fructose-containing beverages such as cola [15]. In our study, while both the cola and the fruit drink contained HFCS as the primary sweetener [30], there was a lower glucose to fructose ratio in the cola. A possible rationale for this seemingly contradictory finding in the present study may be that cola carbonation was a greater factor contributing to FI regulation than its sugars ratio. Beverage carbonation has been shown to have a dose-dependent effect on satiety when consumed 10 min before a meal [31] and carbonation may decrease appetite via an increase in gastric distension [32]. However, a recent study in adult women that used magnetic resonance imaging showed carbonation positively affected gastric volume but did not affect FI or satiety [33]. The effect of gastric distension may be very short term due to rapid absorption of carbon dioxide [33] and may not strongly affect gastric emptying or fluid intake beyond 15 min in healthy adults [34]. Even though it may be possible that carbonation of cola suppressed FI in the present study, further research examining the effect of carbonation on satiety and FI in children is warranted.

Despite the additional 12 g of protein in the chocolate milk drink, no differences were found in FI suppression when directly comparing chocolate milk, cola, and the fruit drink, but the findings are consistent with several reports. No differences in test meal FI were observed between chocolate milk and orange juice when consumed 2 h before an ad-libitum meal in young healthy adults [19]. A more recent study in children aged 9–14 years of age reported that FI was not different 60 min after consuming isocaloric fruit punch or chocolate milk [12]. The milk proteins casein and whey have both previously been shown to enhance satiety and reduce FI in boys [26] and adults [35,36]. In normal weight boys, 50 g of whey protein suppressed FI more than 50 g of glucose after 60 min [26]. In adults, beverages containing whey-protein ranging from 20 to 50 g suppressed FI compared to sucrose, casein, egg albumin, or soy protein [35,37,38]. Casein has also been shown to reduce subsequent FI more than egg albumin or maltodextrin in healthy men [39]. It has been previously suggested that a threshold amount of protein (~30 g) is required to suppress FI and maximize the effect on satiety [40]. However, in adults, 10–20 g of whey protein suppressed FI 30 min following consumption [37] while 5–20 g did not suppress FI over 120 min [41]. Since little research with protein doses <30 g have been performed in children, it is unclear if there is a threshold amount of protein needed to suppress FI in girls. However, previous research in adults suggests both amount and timing are important since protein doses of <20 g may not suppress FI when the beverage is consumed >30 min before meal consumption.

While the foregoing results suggest a greater effect of some SSBs on FI suppression, cumulative FI did not differ among beverages. Similar to our findings, recent work in children aged 9–14 years of age reported cumulative FI did not differ following consumption of 2% milk and fruit punch treatments [12]. In addition, cumulative FI was about 25% higher in overweight/obese children compared with children at a healthy weight [12], which suggests that cumulative FI is more strongly affected by body weight status. Contrary to our findings, higher cumulative energy intakes were observed 30 min following consumption of isovolumetric amounts (500 mL) of SSBs in adults [19]. It is possible that FI regulation is more sensitive to calories consumed as carbohydrates in children as compared to adults [26,42]. Further, girls may have a stronger regulation of FI compared to boys with more restrained eating behaviors in girls being apparent as young as 3–5 years of age [20].

Sweetness is hypothesized to decrease FI [43] and increase feelings of fullness [44] in children. However, beverage sweetness did not strongly predict FI suppression, which suggests that physiological properties of the beverages were stronger determinants of FI suppression. Furthermore, even though palatability has previously been thought to be positively associated with FI in healthy adults [45], short-term FI in the present study was not affected by beverage palatability. Research supporting our findings reported that beverage sweetness and pleasantness of glucose solutions did not associate with FI at a test meal in boys at normal weights [26].

It is also possible that sex differences in expressing subjective appetite exist between boys and girls. Subjective appetite did not decrease in boys 15 min after consuming glucose or HFCS solutions (250 mL, ~200 kcal) [14] while, in the present study, girls reported having suppressed appetites 15 min after consuming all beverage types. It is not clear whether this is due to greater attention to the study protocol by girls compared with boys or if girls experience greater satiety due to greater and sustained activation of satiety centers. However, solid snacks with similar energy contents consumed by children decreased subjective appetite [46], which suggests that children are better able to express changes in subjective appetite from solid compared to liquid foods.

There are several limitations in the present study. We did not include a flat cola and are, therefore, uncertain whether carbonation affected FI suppression. Furthermore, we did not measure satiety hormones, which limits our ability to determine the influence of physiological mechanisms influencing FI regulation. Rest of day FI data was not collected in this study, which limits our ability to understand the effect of SSB intake on energy intake over a 24-h period. We did not explore if between-meal beverage consumption affected FI differently than within meal beverage intake. This is an important outcome for exploration in future studies as early work in women showed caloric beverage consumption with a meal does not affect satiety or suppress FI [47].

## 5. Conclusions

In conclusion, chocolate milk and cola, but not fruit drink, suppressed FI in girls and cumulative FI did not differ among treatments.

## Figures and Tables

**Table 1:** Nutritional composition of beverages ^1^.

	Water	Fruit Drink	Cola	Chocolate Milk
Volume (mL)	350	350	350	350
Calories (kcal)	0	154	158	224
Fat (g)	0	0	0	4
Protein (g)	0	0	0	12
Carbohydrates (g)	0	38	38	38
Total Sugars (g)	0	34	38	37
Sucrose (g)	0	0	0	20
Glucose (g)	0	18	16	-
Fructose (g)	0	16	22	-
Lactose (g)	0	0	0	17
Glucose: Fructose	0	1.1	0.7	-

^1^ Nutritional composition of the beverages was determined by the Maxaam Analytics International Corporation, Mississauga, Ontario following AOAC Methods of Analysis [22].

**Table 2:** Baseline Characteristics.

Variable	Mean ± SEM
Age (year)	11.8 ± 0.3
Body Mass (kg)	49.9 ± 3.1
Height (m)	1.5 ± 0.02
BMI percentile	68.5 ± 6.0
Fat mass ^1^ (%)	23.7 ± 2.0
Fat mass ^1^ (kg)	12.9 ± 1.9
Fat-free mass ^1^ (%)	76.3 ± 2.0
Fat-free mass ^1^ (kg)	37.0 ± 1.9

All values are mean ± SEM, *n* = 28. BMI = body mass index. ^1^ Body composition measures (fat mass, fat free mass) were calculated from the sum of four skinfolds (mm) at triceps, biceps, suprailiac, and subscapular [24].

**Table 3:** Effect of beverages on energy and water intake at 60 min and sweetness and palatability of the beverages and pizza in girls ^1^.

	Beverage			
	Chocolate Milk	Cola	Fruit Drink	Water
FI (kcal)	795 ± 43 *	840 ± 49 *	872 ± 48	942 ± 47
Cumulative FI (kcal)	1019 ± 43	998 ± 49	1026 ± 48	942 ± 47
Caloric compensation (%)	66 ± 13	65 ± 24	46 ± 21	-
Water intake (mL)	203 ± 29	200 ± 33	182 ± 28	187 ± 29
Beverage sweetness (mm)	67 ± 4 *	59 ± 6 *	78 ± 4 *^,‡^	14 ± 4
Beverage palatability (mm)	81 ± 4 *^,‡^	56 ± 6	70 ± 6 *	42 ± 5
Pizza pleasantness (mm)	87 ± 3	86 ± 3	86 ± 3	88 ± 3

^1^ Values are mean ± SEM; *n* = 28. A one-way ANOVA with beverage as the main factor was conducted. FI: food intake; * Different from water at *p* < 0.05 (*Tukey–Kramer’s* test, adjusted for multiple comparisons). ^‡^ Different from cola at *p* < 0.05 (*Tukey–Kramer’s* test, adjusted for multiple comparisons).

## References

[1] Grimm G.C., Harnack L., Story M. (2004). Factors associated with soft drink consumption in school-aged children. J. Am. Diet. Assoc..

[2] Hendel-Paterson M., French S.A., Story M. (2004). Parental attitudes towards soft drink vending machines in high schools. J. Am. Diet. Assoc..

[3] Drewnowski A., Rehm C.D., Constant F. (2013). Water and beverage consumption among children age 4–13 y in the United States: Analyses of 2005–2010 NHANES data. Nutr. J..

[4] Danyliw A.D., Vatanparast H., Nikpartow N., Whiting S.J. (2011). Beverage intake patterns of Canadian children and adolescents. Public Health Nutr..

[5] Chan T.F., Lin W.T., Huang H.L., Lee C.Y., Wu P.W., Chiu Y.W., Huang C.C., Tsai S., Lin C.L., Lee C.H. (2014). Consumption of sugar-sweetened beverages is associated with components of the metabolic syndrome in adolescents. Nutrients.

[6] DeBoer M.D., Scharf R.J., Demmer R.T. (2013). Sugar-sweetened beverages and weight gain in 2- to 5-year-old children. Pediatrics.

[7] Popkin B.M., Armstrong L.E., Bray G.M., Caballero B., Frei B., Willett W.C. (2006). A new proposed guidance system for beverage consumption in the United States. Am. J. Clin. Nutr..

[8] Rivera J.A., Munoz-Hernandez O., Rosas-Peralta M., Aguilar-Salinas C.A., Popkin B.M., Willett W.C. (2008). Beverage consumption for a healthy life: Recommendations for the Mexican population. Rev. Investig. Clin..

[9] Health Canada (2011). Eating Well with Canada’s Food Guide.

[10] Erickson J., Sadeghirad B., Lytvyn L., Slavin J., Johnston B.C. (2017). The scientific basis of guideline recommendations on sugar intake: A systematic review. Ann. Int. Med..

[11] Ma J., Karlsen M.C., Chung M., Jacques P.F., Saltzman E., Smith C.E., Fox C.S., McKeown N.M. (2016). Potential link between excess added sugar intake and ectopic fat: A systematic review of randomized controlled trials. Nutr. Rev..

[12] Vien S., Luhovyy B.L., Patel B.P., Panahi S., El Khoury D., Mollard R.C., Hamilton J.K., Anderson G.H. (2016). Pre-and within-meal effects of fluid dairy products on appetite, food intake, glycemia, and regulatory hormones in children. Appl. Physiol. Nutr. Metab..

[13] Bray G.A. (2008). Fructose: Should we worry?. Int. J. Obes..

[14] Van Engelen M., Khodabandeh S., Akhavan T., Agarwal J., Gladanac B., Bellissimo N. (2014). Effect of sugars in solutions on subjective appetite and short-term food intake in 9- to 14-year-old normal weight boys. Eur. J. Clin. Nutr..

[15] Akhavan T., Anderson G.H. (2007). Effects of glucose-to-fructose ratios in solutions on subjective satiety, food intake, and satiety hormones in young men. Am. J. Clin. Nutr..

[16] Melanson K.J., Zukley L., Lowndes J., Nguyen V., Angelopoulos T.J., Rippe J.M. (2007). Effects of high-fructose corn syrup and sucrose consumption on circulating glucose, insulin, leptin, and ghrelin and on appetite in normal-weight women. Nutrition.

[17] Stanhope K.L., Griffen S.C., Bair B.R., Swarbrick M.M., Keim N.L., Havel P.J. (2008). Twenty-four-hour endocrine and metabolic profiles following consumption of high-fructose corn syrup-, sucrose-, fructose-, and glucose-sweetened beverages with meals. Am. J. Clin. Nutr..

[18] Almiron-Roig E., Drewnowski A. (2003). Hunger, thirst, and energy intakes following consumption of caloric beverages. Physiol. Behav..

[19] Panahi S., Luhovyy B.L., Liu T.T., Akhavan T., El Khoury D., Goff H.D., Anderson G.H. (2013). Energy and macronutrient content of familiar beverages interact with pre-meal intervals to determine later food intake, appetite and glycemic response in young adults. Appetite.

[20] Birch L.L., Fisher J.O. (1998). Development of eating behaviors among children and adolescents. Pediatrics.

[21] Ogden C.L., Kuczmarski R.J., Flegal K.M., Mei Z., Guo S., Wei R., Grummer-Strawn L.M., Curtin L.R., Roche A.F., Johnson C.L. (2002). Centers for Disease Control and Prevention 2000 growth charts for the United States: Improvements to the 1977 national center for health statistics version. Pediatrics.

[22] AOAC (Association of Official Analytical Chemists) (2016). Official Methods of Analysis of AOAC International.

[23] Heymsfield S.B., Williams P.J., Shils M.E., Young V.R. (1988). Nutritional assessment by clinical and biochemical methods. Modern Nutrition in Health and Disease.

[24] Brook C.G. (1971). Determination of body composition of children from skinfold measurements. Arch. Dis. Child..

[25] De Zepetnek J.O.T., Pollard D., Welch J.M., Rossiter M., Faghih S., Bellissimo N. (2017). Pre-meal screen-time activities increase subjective emotions, but not food intake in young girls. Appetite.

[26] Bellissimo N., Desantadina M.V., Pencharz P.B., Berall G.B., Thomas S.G., Anderson G.H. (2007). A comparison of short-term appetite and energy intakes in normal weight and obese boys following glucose and whey-protein drinks. Int. J. Obes..

[27] Birch L., Deysher M. (1985). Conditioned and unconditioned caloric compensation: Evidence for self-regulation of food intake in young children. Learn. Motiv..

[28] Bellissimo N., Thomas S.G., Pencharz P.B., Goode R.C., Anderson G.H. (2008). Reproducibility of short-term food intake and subjective appetite scores after a glucose preload, ventilation threshold, and body composition in boys. Appl. Physiol. Nutr. Metab..

[29] Patel B.P., Bellissimo N., Thomas S.G., Hamilton J.K., Anderson G.H. (2011). Television viewing at mealtime reduces caloric compensation in peripubertal, but not postpubertal, girls. Pediatr. Res..

[30] Hanover L.M., White J.S. (1993). Manufacturing, composition, and applications of fructose. Am. J. Clin. Nutr..

[31] Moorhead S.A., Livingstone M.B., Dunne A., Welch R.W. (2008). The level of carbonation of a sugar-sweetened beverage preload affects satiety and short-term energy and food intakes. Br. J. Nutr..

[32] Passe D.H., Horn M., Murray R. (1997). The effects of beverage carbonation on sensory responses and voluntary fluid intake following exercise. Int. J. Sport Nutr..

[33] Cuomo R., Savarese M.F., Sarnelli G., Nicolai E., Aragri A., Cirillo C., Vozzella L., Zito F.P., Verlezza V., Efficie E. (2011). The role of a pre-load beverage on gastric volume and food intake: Comparison between non-caloric carbonated and non-carbonated beverage. Nutr. J..

[34] Ryan A.J., Navarre A.E., Gisolfi C.V. (1991). Consumption of carbonated and noncarbonated sports drinks during prolonged treadmill exercise in the heat. Int. J. Sport Nutr..

[35] Anderson G.H., Tecimer S.N., Shah D., Zafar T.A. (2004). Protein source, quantity, and time of consumption determine the effect of proteins on short-term food intake in young men. J. Nutr..

[36] Veldhorst M.A., Nieuwenhuizen A.G., Hochstenbach-Waelen A., Westerterp K.R., Engelen M.P., Brummer R.-J.M., Deutz N.E., Westerterp-Plantenga M.S. (2008). Comparison of the effects of a high-and normal-casein breakfast on satiety, ‘satiety’hormones, plasma amino acids and subsequent energy intake. Br. J. Nutr..

[37] Akhavan T., Luhovyy B.L., Brown P.H., Cho C.E., Anderson G.H. (2010). Effect of premeal consumption of whey protein and its hydrolysate on food intake and postmeal glycemia and insulin responses in young adults. Am. J. Clin. Nutr..

[38] Boirie Y., Dangin M., Gachon P., Vasson M.P., Maubois J.L., Beaufrere B. (1997). Slow and fast dietary proteins differently modulate postprandial protein accretion. Proc. Natl. Acad. Sci. USA.

[39] Lorenzen J., Frederiksen R., Hoppe C., Hvid R., Astrup A. (2012). The effect of milk proteins on appetite regulation and diet-induced thermogenesis. Eur. J. Clin. Nutr..

[40] Leidy H.J., Clifton P.M., Astrup A., Wycherley T.P., Westerterp-Plantenga M.S., Luscombe-Marsh N.D., Woods S.C., Mattes R.D. (2015). The role of protein in weight loss and maintenance. Am. J. Clin. Nutr..

[41] Poppitt S.D., Proctor J., McGill A.-T., Wiessing K.R., Falk S., Xin L., Budgett S.C., Darragh A., Hall R.S. (2011). Low-dose whey protein-enriched water beverages alter satiety in a study of overweight women. Appetite.

[42] Birch L.L., Deysher M. (1986). Caloric compensation and sensory specific satiety: Evidence for self regulation of food intake by young children. Appetite.

[43] Lavin J.H., French S.J., Ruxton C.H., Read N.W. (2002). An investigation of the role of oro-sensory stimulation in sugar satiety?. Int. J. Obes..

[44] Birch L.L., McPhee L., Sullivan S. (1989). Children’s food intake following drinks sweetened with sucrose or aspartame: Time course effects. Physiol. Behav..

[45] Van Wymelbeke V., Beridot-Therond M., de La Gueronniere V., Fantino M. (2004). Influence of repeated consumption of beverages containing sucrose or intense sweeteners on food intake. Eur. J. Clin. Nutr..

[46] Patel B.P., Luhovyy B., Mollard R., Painter J.E., Anderson G.H. (2013). A premeal snack of raisins decreases mealtime food intake more than grapes in young children. Appl. Physiol. Nutr. Metab..

[47] DellaValle D.M., Roe L.S., Rolls B.J. (2005). Does the consumption of caloric and non-caloric beverages with a meal affect energy intake?. Appetite.

